# General Therapeutics and Materia Medica, Adapted for a Medical Text-Book

**Published:** 1851-01

**Authors:** 


*General Therapeutics and Materia Medica,* adapted for a Medical Text-Book. By ROBLEY DUNGLISON, M. D., etc., etc., etc. One hundred and eighty-two illustrations. Fourth edition, revised and improved. In two volumes. Philadelphia; LEA & BLANCHARD. 1850.

In this new edition of a work sufficiently familiar, remedial agents of recent introduction have been added; the number of illustrations have been greatly increased, and a copious index of diseases and remedies appended.

These improvements entitle it to continued favor.

The general tone of the author's remarks on General Therapeutics is eminently in the spirit of the modern practice of medicine.

For sale by Phinney & Co., and Derby & Co.

